# Some Conditions under Which Infants Are Nursed Away from Home

**Published:** 1908-05-16

**Authors:** Alfred Greenwood

**Affiliations:** Medical Officer of Health for Blackburn


### SPECIAL ARTICLE.

### SOME CONDITIONS UNDER WHICH INFANTS ARE NURSED AWAY FROM HOME.

By ALFRED GREENWOOD, M.D., D.P.H., Medical Officer of Health for Blackburn.

It is generally conceded that one of the most important problems of preventive medicine is the amelioration of the conditions under which young infants are fed and nursed, with the object of reducing the number of preventable deaths below the age of one year. And it should not be forgotten that a reduced infantile death-rate implies a reduced sickness rate. Death-rates are sad figures in themselves, but they by no means record the whole story, since they take no account of the maimed and wrecked lives resulting from illnesses which have not ended fatally.

Numerous articles have appeared in the lay and medical press, papers have been read at various conferences, and experiments have been tried in certain towns by public-spirited citizens to remedy what defects they can in our social organisation. The number of side-issues into which the student of infantile mortality is led is legion. In this short communication I shall endeavour to draw attention to some conditions under which infants are nursed away from their own homes during the daytime whilst their mothers are at work in the mills. The term infant in this article refers to children below the age of one year.

This is a sub-section of the problem of infantile mortality which has a special interest in Blackburn. The Infant Life Protection Act of 1897 is an Act to amend the law for the better protection of infant life, and it repealed the Infant Life Protection Act of 1872. The 1897 Act makes it compulsory on every person retaining or receiving for hire or reward in that behalf more than one infant under the age of five years for the purpose of nursing or maintaining such infants apart from their parents for a longer period than forty-eight hours, to give notice thereof to the local authority within the forty-eight hours. Other provisions of this Act relate to notification of removal of infants, inspection of infants and premises, character of person in charge of such infants, notification of death to the coroner, etc. There are many defects in this Act, which I have pointed out on a previous occasion.* But the class of infant to which I now refer is not met by the above-mentioned Act. It is well known that in many of the manufacturing towns of Lancashire it is the custom to take the babies in the early morning to be nursed during the day whilst the mother is absent at the mill, and then to bring them back on the mother's return in the evening. Such children, therefore, are not apart from their parents for a longer period than 48 hours. Also the Infant Life Protection Act does not contain any provision for regulating the placing out to nurse of single children.

The distances which these infants are carried twice daily, the amount of clothing in which they are wrapped for the journeys, the character and age of the woman in charge, the kind of house, the quality and quantity of food, vary considerably. For the sake of the infant it is most undesirable that this possibility of variation should exist. However, so long as young married women work in the mills, either from choice or compulsion, so long must some provision be made for their infants to be nursed during the day; and it should be our duty to consider and take such steps as will ensure that these infants shall be looked after under the best conditions possible.

It may be said that the situation could be met by the provision of crèches. It is true that these institutions have their advantages, but they are certainly not without considerable disadvantages. A large number would be needed in various parts of the town, and the difficulties regarding the spread of infectious diseases amongst collections of infants might be great. Moreover, if crèches were established the mothers could not be compelled to take their babies to them. Indeed, I believe that some years ago three crèches were established in Blackburn as private ventures, but that they proved failures, because the mothers would not use them.

Some idea of the acuteness of this question in Blackburn may be gleaned from the fact that out of a total population of 127,626 at the 1901 census about 3,148 married women work in the mills, and that there are about 3,000 births each year. About 500 infants are regularly taken away from their own homes to be nursed during the day in other houses.

Of the boarded-out children, of whom particulars have been obtained from January to October 1907, the mothers returned to work as follows:—

| | | | |
|---|---|---|---|
| 1 mother | returned to work | 2 days | after confinement. |
| 1 " | " | 3 days | " |
| 1 " | " | 1 week | " |
| 14 mothers | " | 3 weeks | " |
| 124 " | " | 1 month | " |
| 178 " | " | between 1 and 2 months after confinement. | |

The rest returned after 4 months.

In April 1907 two lady sanitary inspectors commenced work in Blackburn under the supervision of the medical officer of health in inspecting infants and in giving instructions as regards their feeding and management. Since their appointment they have paid nearly 3,000 visits to houses where births have occurred, and 1,021 re-visits in order to ascertain whether their instructions had been carried out or not. These two ladies are trained nurses, certified midwives, and certificated sanitary inspectors. The great advantages of this triple qualification are obvious, inasmuch as most useful help can be given in nursing infants, inspecting midwives, and reporting sanitary defects which require remedies.

At first, owing to prejudice, there was considerable resentment on the part of some mothers in having their children inspected. To some extent, owing to the tact and patience of these two officials, this maternal objection has disappeared, and in certain instances the visit of the inspector is looked for, and advice is asked for, whereas previously it was refused or ignored. This improvement, however, applies, for the most part, to the more intelligent women. Many of those women who require most teaching are the least teachable and accessible. The latter group, after repeated advice to the contrary, will persist in using long-tubed feeding-bottles, bread and milk, cheap patent foods, and "anything that is going" for feeding the babies who are under their temporary charge, and, in general, neglecting to do what they should do. It is therefore clear that ignorance and prejudice are still formidable factors to combat.

Infants who are nursed away from home during the day are taken about half-past five every morning, wrapped in a shawl or blanket, to the care of a woman, frequently beyond middle age, and frequently unable to look after any child properly. Similarly they are carried home again about 6 o'clock in the evening. About five shillings a week is the sum paid to this woman, out of which she provides milk for the child. Sometimes she takes another child, two or three years old, for whom she receives an additional sum of four shillings weekly.

The character of many of these women and their occupations vary. Some of them are untidy in dress, coarse in language, and careless and intemperate in habits. Others, of course, are the reverse. In the hands of an unscrupulous woman, to whom a certain sum of money has been paid by the mother for the purchase of milk, it is possible that an infant might be kept short of food. I have not met any case of this kind, but only mention the possibility. I am sure, however, that condensed milk is often used as a substitute for fresh cow's milk, when it should not be so used. At many of these houses the milkman only calls once a day—between 8 and 10 o'clock in the morning—so that the infant is in its temporary residence two hours without fresh milk. Also the milk is frequently kept uncovered in a dusty place.

Many of the babies change hands periodically. The mother, for various reasons, may "have a few words" with the woman in charge, and the baby is subsequently lodged in another domicile. Sometimes, after the lady inspector has remonstrated with the woman in charge, the latter has given up the infant, who has again been housed elsewhere.

* Paper read before the National Conference on Infantile Mortality in London, 1906.

The following are particulars respecting certain infants who are nursed away from home, and who have been visited by the lady sanitary inspectors:—

1. E.G. (mother, weaver).—Mother commenced work at the mill two months after birth of infant, and during this period the infant was fed naturally altogether. When the infant was two months old it was taken each morning a short distance to be nursed by a woman who was satisfactory, but the child was nursed here for six weeks only. After this the infant was carried by the mother a distance of about one mile and a half each morning in order that she might be looked after in another house by the maternal grandmother, who is not satisfactory.

2. M. E. (cardroom hand).—Resumed work when the infant was one month old, during which time it was breast-fed entirely. It was then taken a distance of about half a mile to be looked after by the maternal grandmother, who ignored the lady inspector's instructions, and fed the child upon bread, which resulted in diarrhœa.

3. E. H. (mother, weaver).—Resumed work two months after the birth of the infant, during which time it was partially breast-fed. The infant was then taken a short distance each morning to be nursed by a woman who followed the lady inspector's instructions, and who refused, in accordance with these instructions, to give the infant bread. The mother on this account took the child away and put her in charge of another woman who was very unsatisfactory and who ignored all instructions.

4. L. P. (mother, weaver).—Resumed work five weeks after birth of infant. It was then nursed near its own home by a woman who was extremely unsatisfactory, and who eventually was taken to the asylum. The infant is now nursed at another house in the immediate neighbourhood of its own home, under more satisfactory conditions.

5. J. C. (mother, cardroom hand).—Resumed work when the infant was one month old, and during which time it was fed partly by the breast. It was then taken a distance of about a quarter of a mile, where it was nursed very unsatisfactorily by a girl aged seventeen years. After about a month the infant was taken away from this house and carried a distance of about one and a half miles night and morning, where the conditions were anything but satisfactory regarding the nursing and feeding of the infant.

6. R. H. (mother, weaver).—Resumed work six weeks after the birth of the child. This infant was fed on dry sweet biscuits when it was six weeks old. It was then taken to an adjoining house, where it was again fed contrary to the instructions given.

7. E. F. (mother, weaver).—Infant nursed at first near its own home. When visited on April 18, nurse and house were clean, but nurse insisted on giving bread and milk, and refused to alter this mode of feeding. On July 19 the infant was being nursed at home, partially breast-fed and partly on bread and milk. On September 14 the infant was being nursed at another house, near its own home, and was still being fed on bread and milk.

8. L. B.—Dirty, shiftless mother and dirty house. Eyes of another child in same house were very inflamed. Infant was fed from a long-tube bottle, which was dirty. Child was very puny. On September 24 the mother was again at work, and the child was being nursed next door. When examined, the baby was verminous and dirty and fed in the old way.

9. J. W.—Born February 22; illegitimate child. Mother lives with her aunt, but sometimes is away at work. About twelve people live in the house. Infant fed on rusks and on milk and water from long-tubed bottle. Visited repeatedly. Condition of infant always unsatisfactory. The woman was warned each time, but would not alter. Infant in a wasted condition on September 23, weighing about seven pounds—that is, about half the weight it should have been. It died on November 15.

10. J. S. A.—Fed with milk and water and Mellin's Food from a long-tubed bottle. Husband and wife lodging here. Tenant old woman and suspected of alcoholism. Mother very careless. Clothes of child and cradle damp. General neglect. At the next visit the conditions were better and a boat bottle was being used. The father and mother separated, but mother removed to another house. When visited there, the inspector found condition dirty and infant fed on milk and water from long-tubed bottle.

11. F. C.—Born February 12. Mother aged eighteen years, lived in lodgings. Father said to be lazy. Mother removed to infirmary. Infant was first nursed near its own home and fed on milk and water from a long-tubed bottle. At the next visit the infant was nursed at another house in the neighbourhood, and, when visited later on still, at another house, with the parents of the father. Again nursed at another house. Later on was nursed at one of the above-mentioned houses again. There was also another infant nursed at this house, which was dirty. Two old women looked after these two infants. Both these women were unsatisfactory. After repeated visits and advice there was no improvement. One of the infants is now wasting.

12. A. G.—Born December 13; illegitimate. Mother aged nineteen years. Fed on Nestlé's Milk from a long-tubed bottle. When visited on May 8, the inspector was told that the infant had bronchitis, whooping-cough, and consumption of the bowels. This infant was next visited at another house in the same street. No improvement was made in the feeding. The inspector remonstrated strongly, and when she visited next she found the infant at still another house being fed most unsatisfactorily.

In other cases where an infant is put out to nurse during the day the outlook is certainly brighter. These infants, when coming under the care of an intelligent and careful woman of an amiable disposition appear to be as well cared for as they are at home. The temporary guardian in such cases baths the infant every morning, attends carefully to the quality and quantity of food, and to the mode of its administration, in a manner deserving of the highest praise.

The greatest difficulty we have experienced in Blackburn with these temporary nurses has been in respect to the feeding of the infants. The two lady inspectors have visited houses and found the woman in charge feeding an infant of three months of age on bread and milk, milk in a dirty long-tubed bottle, biscuits, or even worse. The inspector has pointed out to the woman that such a young infant cannot digest starchy foods, and that fermentation must occur in the stomach if dirty milk or improper food is used. The woman has promised to follow out suitable instructions as a result of this visit. At a subsequent visit the woman is frequently found feeding the infant in the old and improper way. On remonstrating with her, such answers as these are received: " The child's mother said it was to have bread, and I shall take my instructions from her and not from you," or " I have had eleven children and fed them in my own way " (sometimes it transpires that she buried ten of them). Occasionally, the inspector has been told plainly that she was not wanted to visit. Cases like these have occurred over and over again. Considerable difficulty has also been experienced in getting these women to scald the feeding-bottles, and some of them prefer to feed an infant three or four months old at intervals during the day with a mixture of bread and milk, kept in a cup on the top of the oven.

Other difficulties which have been experienced in the inspection of these infants temporarily boarded out have been in reference to cleanliness and clothing. Proper baths are seldom given to these children. A common method of so-called cleansing is to lather them with soap, which the woman attempts to wash off ineffectively with water from a small can. Precautions are not taken against the infant catching cold during this operation. I have referred previously to the lack of cleanliness in the food utensils. As a rule, there has been less need to find fault with the small amount of clothing, but even this is not always satisfactory. Unclean cradles and damp clothing have been found in some cases. The infants, however, are exposed to great changes of temperature, which certainly is a prolific cause of bronchitis. This need not occasion surprise when it is remembered that an infant is taken from a warm bed and carried a varying distance through the cold street in the early morning, and that this act is repeated at night. I am unable to say in what way this can be avoided. All that can be done in this respect is to advise mothers to wrap them up as thoroughly as possible, when they are taken out night and morning, so long as they have to be taken out. This same objection would also apply to crèches. Another difficulty which has been experienced refers to the failure, on the part of the woman in charge, to inform the mother of the lady inspector's instructions.

It is clear, therefore, from what I have stated above, that some change in procedure would be a great help. In seeking for a remedy which might be suggested, one is confronted with many difficulties. Actual direct compulsion in the correct feeding of these infants would be impracticable in a large town such as this; also over-legislation should be avoided. We can only hope that many of these dangerous prejudices will disappear in time as knowledge spreads. I am afraid, however, that this disappearance, in many cases, will be synchronous only with the termination of the existence of this mischievous type of Mother Gamp. It is surprising how often young mothers are influenced by certain grandmotherly practices, to the detriment of their infants. At the same time, it is worthy of consideration whether or not all women having charge of these infants during the daytime should be compelled to register their names and addresses at the Health Office. This would indicate that the Municipality took cognisance of such women, and would give it the opportunity, with a sufficient and suitable staff of ladies, of visiting and revisiting, at frequent intervals, these infants thus temporarily boarded out. If, in addition, the Municipality had the power to erase the name from this register of any woman found incapable of, or unsuitable for, this duty, and to prevent her, on penalty of a fine, from taking charge of infants again, a great lever would be placed in the hands of those who are anxious to reduce the infantile mortality rate.

Women who were doing their duty and were anxious for the welfare of the infants under their charge would not object to registration and inspection. As I have hinted before, I am doubtful whether many of these women who assume the rôle of temporary nurses and guardians of infants will ever show much improvement. A few will, perhaps, improve, and, of course, our present efforts should be continued with unabated vigour. At the same time, in connection with such efforts, I believe great hopes for the future lie in two directions, namely:—

(1) Training the older girls in the public elementary schools in the first principles of hygiene, special stress being laid on the feeding and care of infants. These girls will be mothers themselves some day, and if this desirable knowledge could be inculcated into their minds at a receptive and susceptible age, they would be less likely to be influenced by dangerous prejudices when they have children of their own, or when they have other mothers' children to look after.

(2) An improved type of midwife is a very necessary factor. In Blackburn a considerable number of women at confinement are attended by midwives without the presence of any medical man. The mother, under these conditions, is influenced for good or for evil, especially at the birth of her first child. There can be no doubt that if these women at childbirth could receive the attention of a clean, intelligent midwife they would receive much benefit, and there is no doubt whatever that the baby would receive an excellent start during the first two weeks of life, and the mother, when she went to work four or five weeks later, would be more likely to insist upon the right kind of woman to look after her baby whilst she is at work in the mill.

It is also worthy of consideration whether or not a number of ladies could be found who would undertake voluntarily to visit and revisit certain homes where births have occurred. I think it would be necessary for this work to be carried out in connection with the Public Health administration of the town, and it is very important that all instructions given to mothers should be upon a uniform basis. I feel convinced that in this direction lies a possibility for great good.

